# Artificial Intelligence in Endodontics—A Bibliometric Analysis

**DOI:** 10.1111/aej.70080

**Published:** 2026-04-21

**Authors:** Eric Chen, Justin Jun‐Young Eun, Alan Chen, Sepanta Hosseinpour, Hossein Mohammad‐Rahimi, Ove Andreas Peters, Philip Yuan‐Ho Chien

**Affiliations:** ^1^ School of Dentistry The University of Queensland Herston Queensland Australia; ^2^ Department of Dentistry and Oral Health Aarhus University Aarhus Denmark; ^3^ Department of Conservative Dentistry, Periodontology and Digital Dentistry, LMU University Hospital LMU Munich Munich Germany

**Keywords:** artificial intelligence, bibliometrics, deep learning, endodontics

## Abstract

This study aims to map and evaluate research trends in artificial intelligence applied to endodontics using bibliometric methods. A comprehensive literature search was conducted in PubMed, EMBASE, and Scopus in April 2025 to identify studies addressing artificial intelligence in endodontic diagnosis, treatment, and education. Titles, abstracts, and full texts were screened according to predefined criteria. Bibliometric indicators including publication output, authorship networks, institutional and country contributions, citation patterns, and keyword co‐occurrence were analysed using bibliometric software. 220 studies were included. Research output increased markedly over time, with major contributions from China, the United States, Turkey and India. Collaboration was largely geographically clustered, with limited international integration. Recent publications showed a strong focus on deep learning approaches for diagnostic imaging, particularly cone‐beam computed tomography and image segmentation. Artificial intelligence research in endodontics is expanding rapidly, but broader collaboration and diversification beyond radiographic applications are needed to support clinical translation.

## 1 Introduction

Artificial intelligence (AI) has rapidly emerged as a transformative force in healthcare, encompassing a broad range of computational techniques designed to perform tasks that typically require human intelligence. Within this hierarchy, machine learning represents a subset of AI in which algorithms learn patterns from data, while deep learning constitutes a further subset of machine learning that employs multilayered neural networks to model complex, non‐linear relationships. In dentistry, and particularly in endodontics, the application of AI is especially promising due to procedural complexity, anatomical variability, and the heavy reliance on imaging for diagnosis and treatment planning. Deep learning models have been increasingly used for tasks such as the detection of periapical pathology on cone‐beam computed tomography scans and the assessment of complex root canal morphology on panoramic radiographs. These developments signal a paradigm shift from exclusively clinician‐driven image interpretation towards hybrid workflows that integrate clinician expertise with AI‐assisted decision support, with the potential to enhance diagnostic consistency and clinical efficiency [1].

While AI adoption for image analysis is increasing across dental specialities, endodontics presents distinct challenges and opportunities. Root canal systems often display high anatomical variation; outcome prediction remains difficult, and imaging modalities (2D and 3D) generate large volumes of data that require specialist interpretation. Recent narrative and scoping reviews have catalogued AI tools for tasks such as root canal segmentation, vertical root fracture detection and apical pathology identification. Modern implementations of AI in endodontics include, but aren't limited to, the assessment of root morphology [2], detection of root fractures from radiographs [3, 4], periapical lesion detection [5] and prediction of root canal treatment outcome [6]. For instance, Umer et al.'s critical review described how machine‐learning and deep‐learning algorithms are being used in endodontics to address diagnostic and prognostic tasks [7]. However, the literature remains somewhat fragmented in terms of study design, imaging modality, clinical utility and translational uptake into practice [1].

From a research‐mapping perspective, bibliometric analyses provide a powerful means of quantifying the evolution of a field: they can identify publication trends, authorship networks, institutional collaboration patterns, and thematic evolution over time. In the broader dental domain, bibliometric reviews have already highlighted the rapid expansion of AI research. For example, Zatt et al. [8] analysed AI in dentistry publications from 2000 to 2023. However, when one focuses specifically on AI in endodontics, there is a relative paucity of comprehensive bibliometric work. Although a recent publication by Sevencan & Yavşan 2025 [9], conducted a preliminary scientometric evaluation, they included only 40 articles and relied on a single database (Web of Science) and only examined studies up until March 2024. Accordingly, there are no larger‐scale, multi‐database bibliometric mappings of AI in endodontics that comprehensively chart authorship, institutional and country productivity, keyword dynamics, journal performance and thematic shifts.

Accordingly, a comprehensive multi‐database mapping of AI in endodontics is still lacking. Hence, this study aimed to analyse the evolution of AI‐focused research in endodontics by assessing global publication trends, authorship networks, institutional and international collaboration, influential journals, keyword co‐occurrence and citation performance. By identifying dominant clusters and emerging themes, shifting from early ‘computer‐aided diagnosis'’ concepts to current interests in ‘deep learning’, ‘CBCT segmentation'’ and ‘educational applications’, this analysis provides a better understanding of future research directions.

## 2 Methodology

### 2.1 Search Strategy and Selection of Sources

**FIGURE 1:**
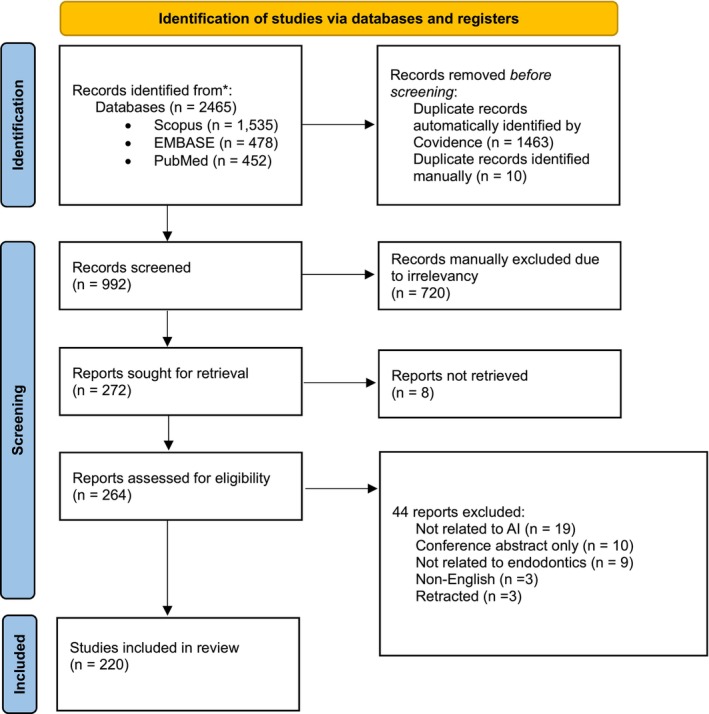
Flowchart showing the screening following the Preferred Reporting Items for Systematic reviews and Meta‐Analyses guidelines (PRISMA).

A comprehensive literature search was conducted in PubMed, EMBASE and Scopus on 30th April 2025 (Figure 1). The search strategies for PubMed, EMBASE and Scopus can be found in Table S1.

Three researchers (AC, EC, and JE) independently screened the collected studies. The inclusion criteria were original research focusing on the applications of AI in endodontic fields as defined by the American Association of Endodontists' Glossary of Endodontic Terms, which is *‘the branch of dentistry concerned with the morphology, physiology and pathology of the human dental pulp and periradicular tissues. Its study and practice encompass the basic and clinical sciences including the biology of the normal pulp and the etiology, diagnosis, prevention and treatment of diseases and injuries of the pulp and associated periradicular conditions*'*’*. The exclusion criteria were non‐English language, studies not related to AI or the field of endodontics, secondary research, and inaccessible studies. An initial title and abstract screening were performed, followed by a full‐text screening. A final screening was subsequently done by a senior investigator (PC) to resolve any conflicts.

### 2.2 Data Extraction and Analysis

Data extracted from each study included the author(s), title, keywords, country of origin, affiliated institution, citations and publication date. The screening and filtering process was completed manually using Covidence (Melbourne, Victoria, Australia). The data were exported in .bib for visualisation using VOSviewer (Version 1.6.20; Centre for Science and Technology Studies, Leiden University, Leiden, The Netherlands) and similarly the .bib format was suitable for use in Bibliometrix (R package Version 4.2.1, University of Naples Federico II, Naples, Italy).

VOSviewer was used to visualise keyword occurrence. Node size and connector thickness represent the number of occurrences and the strength of the cooccurrence link. Where appropriate, full‐counting methods were used over fractional‐counting. Strength of association was defined as ‘the proportion of total co‐occurrences between items to the expected total co‐occurrences between those items'’ [10]. Bibliometrix collated other bibliometric data, including article publication and citation metrics over time, individual author, institution, journal, and country production. Although publication data was sourced from Scopus, EMBASE and PubMed, the metadata for citation analysis is used by Bibliometrix via Scopus only, as PubMed and EMBASE do not provide the metadata for this analysis (https://www.bibliometrix.org/home/index.php/faq). These findings were visualised using Bibliometrix through a series of tables and graphs.

## 3 Results

**FIGURE 2:**
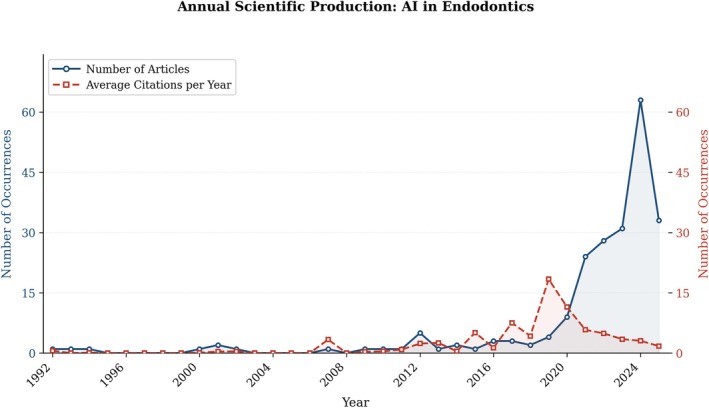
Articles published and average citations per year related to artificial intelligence in endodontics published per year from 1992 to April 2025.

A total of 220 publications met the inclusion criteria for bibliometric analysis. From the 220 studies total, there was only a small selection of studies identified from 1992 to 2015. Publication output was low from 2016 to 2019; however, publication growth noticeably increased from 2020 and with a peak in 2024, with 63 publications. 2025 had a lower number of studies, as this data was collected only up until April 2025 (Figure 2).

### 3.1 Authors

**FIGURE 3:**
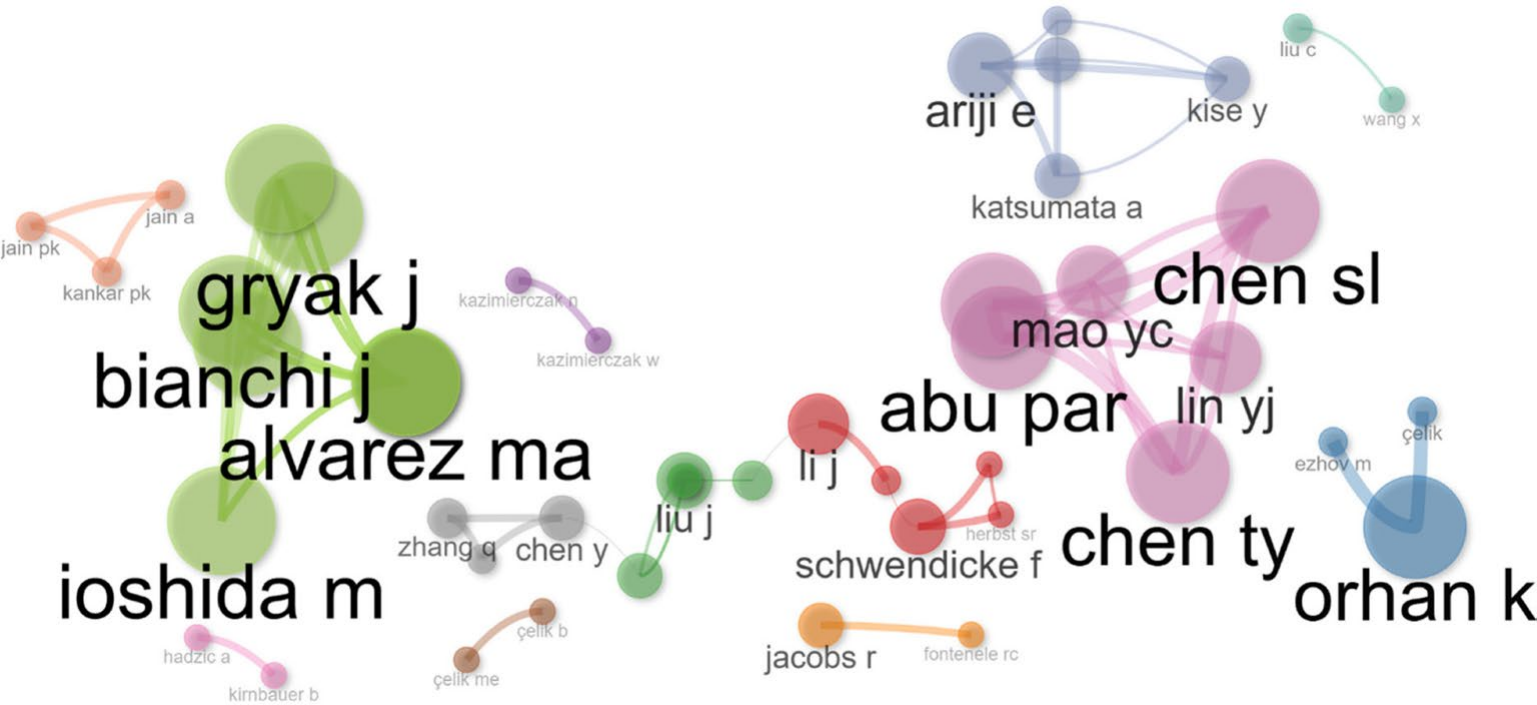
Visualisation of collaborative networks between authors. Colours indicate clustering, while the size of nodes and the thickness of connections represent the amount and strength of collaboration, respectively.

The largest interconnected authorship networks were the groups by Antonia Alvarez Maria and Patricia Angela R Abu. An additional 11 clusters were identified with several established researchers, such as Falk Schwendicke and Kaan Orhan, collaborating predominantly within limited groups (Figure 3).

Orhan, K and Li, J had the highest number of overall publications with 9 and 7 total publications, respectively (Table 1). Orhan, K had large spikes in production in 2022 and 2024, whilst Jacobs, R had a high proportion of her publications in 2025 (Table 2). However, if measured by articles fractionalised (equal splitting credit among all co‐authors), Latke, V and Narawade, V both had the highest fractionalised scores with 1.50 each, despite only having published 3 articles each. In terms of citation counts since publication, Schwendicke, F, Ariji, E, and Orhan, K all had publications with over 200 lifetime citations with accompanying high total citations per year (TCpY).

**TABLE 1:** Total publication counts of authors ranked in descending order of articles published.

Author	Articles	Articles fractionalised
Orhan, K	9	1.25
Li, J	7	1.04
Jacobs, R	6	1.21
Li, S	6	0.94
Liu, C	5	0.63
Liu, J	5	0.92
Schwendicke, F	5	1.07
Abu, PAR	4	0.34
Ariji, E	4	0.53
Chen, CA	4	0.34

**TABLE 2:** Author production over time. The top 10 most prolific authors ranked in descending order by total citations are shown with the authors against publication year.

Author	Year	Number of publications	Total citations	Total citations per year
Schwendicke, F	2019	1	256	36.571
Ariji, E	2019	1	212	30.286
Orhan, K	2020	1	212	35.333
Ariji, E	2020	1	185	30.833
Li, J	2020	1	151	25.167
Li, S	2007	2	128	6.737
Orhan, K	2021	1	107	21.400
Liu, J	2022	2	86	21.500
Li, S	2022	1	85	21.250
Orhan, K	2022	2	71	17.750

### 3.2 Institutions and Countries

**FIGURE 4:**
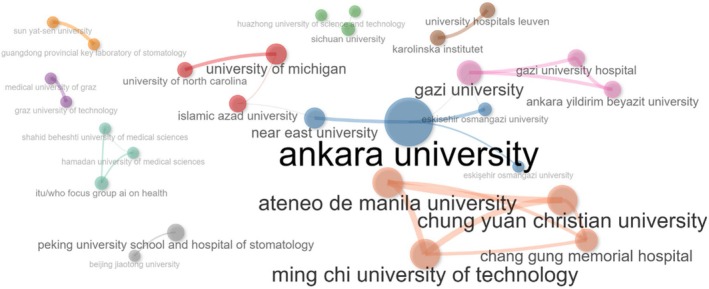
Network of collaboration between institutions. Each node represents a separate institution, whilst the colour and size of the node represent cluster and significance within the cluster. The thickness of the connector represents the strength of collaboration.

Collaboration between institutions was shown but largely confined to the same geographic region, with minimal cross‐regional collaboration. Ten clusters of institutional collaboration are identified (Figure 4). Prominent contributors include Ankara University (Turkey) and Chung Yuan Christian University (Taiwan), with 16 and 9 publications, respectively (Table S2).

**FIGURE 5:**
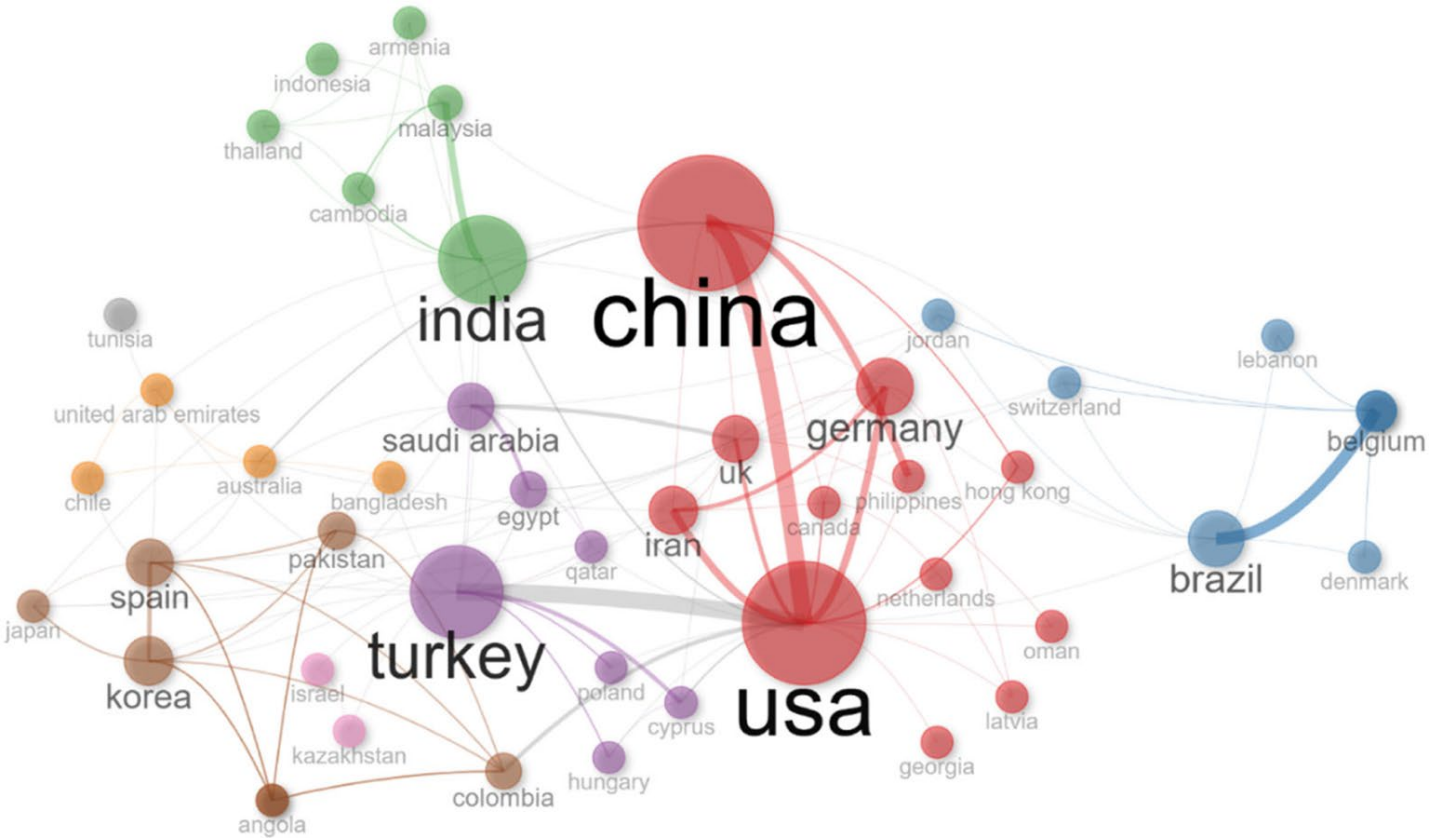
The network of collaboration between countries. Each node represents a separate country, whilst the colour and size of node represent cluster and significance within the cluster. Thickness of connector represents the strength of collaboration.

China, the United States and Turkey are emerging as the most active contributors to globally collaborative research, with significant collaboration between the three locations. There were 3 clusters identified, with China, the United States and Turkey forming a cluster likely due to their large research output. Smaller clusters exist, where India, Malaysia, Thailand and Indonesia form a cluster reflecting their cultural and geopolitical proximity (Figure 5).

Among the top three producers: China, Turkey and India, single country publications (SCPs) outnumber multiple country publications (MCPs), with all three presenting a similar percentage of SCP to MCP (around 22%). Interestingly, Germany is represented solely through SCPs, which indicates there were no international co‐authored publications within the present dataset. In all countries but the US, Belgium and Canada, publications were more often conducted domestically than internationally. China had the highest number of articles produced (*n* = 44), followed by Turkey (*n* = 21) and India (*n* = 19) (Table 3).

**TABLE 3:** Total numerical count of articles per country along with percentage share (%), single country publications and multiple country publications.

Country	Number of articles (%)	Single country publications	Multiple country publications
China	44 (20.0)	34	10
Turkey	21 (9.5)	16	5
India	19 (8.6)	15	4
Germany	10 (4.5)	10	0
USA	10 (4.5)	3	7
Iran	8 (3.6)	4	4
Brazil	7 (3.2)	5	2
Korea	7 (3.2)	5	2
Poland	6 (2.7)	4	2
Saudi Arabia	5 (2.3)	3	2
Spain	5 (2.3)	3	2
Belgium	4 (1.8)	0	4
Indonesia	4 (1.8)	4	0
Japan	4 (1.8)	2	2
Denmark	3 (1.4)	2	1
Egypt	3 (1.4)	2	1
Pakistan	2 (0.9)	2	1
Australia	2 (0.9)	1	1
Austria	2 (0.9)	2	0
Canada	2 (0.9)	0	2

### 3.3 Journals

The *Journal of Endodontics* ranked the highest with 20 publications, followed by *Dentomaxillofacial Radiology* and the *Journal of Dentistry*, each with 10 publications (Table 4). *Journal of Endodontics* also had the highest h‐index of 9 compared to the *International Endodontic Journal*, which had an h‐index of 5, being the only other endodontics‐specific journal in the top 10 journal rankings.

**TABLE 4:** The Top 25 cited journal articles ranked in descending order by total citations.

Paper (First Author, Year, Journal)	DOI	Total citations	TC per year	Normalised TC
Ekert, T, 2019, J Endod [11]	10.1016/j.joen.2019.03.016	256	36.57	1.99
Hiraiwa, T, 2019, Dentomaxillofac Radiol [2]	10.1259/dmfr.20180218	212	30.29	1.65
Orhan, K, 2020, Int Endod J [12]	10.1111/iej.13265	212	35.33	3.09
Fukuda, M, 2020, Oral Radiol [3]	10.1007/s11282‐019‐00409‐x	185	30.83	2.69
Setzer, FC, 2020, J Endod [13]	10.1016/j.joen.2020.03.025	151	25.17	2.20
Johari, M, 2017, Dentomaxillofac Radiol [14]	10.1259/dmfr.20160107	120	13.33	1.77
Ezhov, M, 2021, Sci Rep [15]	10.1038/s41598‐021‐94 093‐9	107	21.40	3.71
Li, S, 2022, J Dent [16]	10.1016/j.jdent.2022.104107	85	21.25	4.34
Saghiri,MA, 2012, Int Endod J [17]	10.1111/j.1365‐2591.2011.01970.x	84	6.00	2.53
Duan, W, 2021, Dentomaxillofac Radiol [18]	10.1259/dmfr.20200251	72	14.40	2.49
Yilmaz, E, 2017, Comput Methods Programs Biomed [19]	10.1016/j.cmpb.2017.05.012	67	7.44	0.99
Yang, J, 2018, Proc Int Comput Software Appl Conf [20]	10.1109/COMPSAC.2018.00076	66	8.25	1.94
Suárez, A, 2024, Int Endod J [21]	10.1111/iej.13985	66	33.00	10.74
Saghiri, MA, 2012, J Endod [22]	10.1016/j.joen.2012.05.004	66	4.71	1.99
Li, S, 2007, Pattern Recogn [23]	10.1016/j.patcog.2007.01.012	64	3.37	1.00
Khan,HA, 2021, Oral Surg Oral Med Oral Pathol Oral Radiol [24]	10.1016/j.oooo.2020.08.024	63	12.60	2.18
Okada, K, 2015, Med Phys [25]	10.1118/1.4914418	56	5.09	1.00
Pauwels, R, 2021, Oral Surg Oral Med Oral Pathol Oral Radiol [26]	10.1016/j.oooo.2021.01.018	52	10.40	1.80
Bayrakdar, IS, 2022, Biomed Res Int [27]	10.1155/2022/7035367	52	13.00	2.65
Li, CW, 2021, Sensors [16]	10.3390/s21217049	49	9.80	1.70
Jeon, SJ, 2021, Dentomaxillofac Radiol [28]	10.1259/dmfr.20200513	47	9.40	1.63
Mohammad‐Rahimi, H, 2024, Int Endod J [1]	10.1111/iej.14014	46	23.00	7.49
Sherwood, AA, 2021, J Endod [29]	10.1016/j.joen.2021.09.009	45	9.00	1.56
Leo, L, 2021, Microprocessors Microsyst [30]	10.1016/j.micpro.2021.103836	40	8.00	1.39
Lin, X, 2021, J Endod [31]	10.1016/j.joen.2021.09.001	39	7.80	1.35

### 3.4 Keywords and Thematic Analysis

**FIGURE 6:**
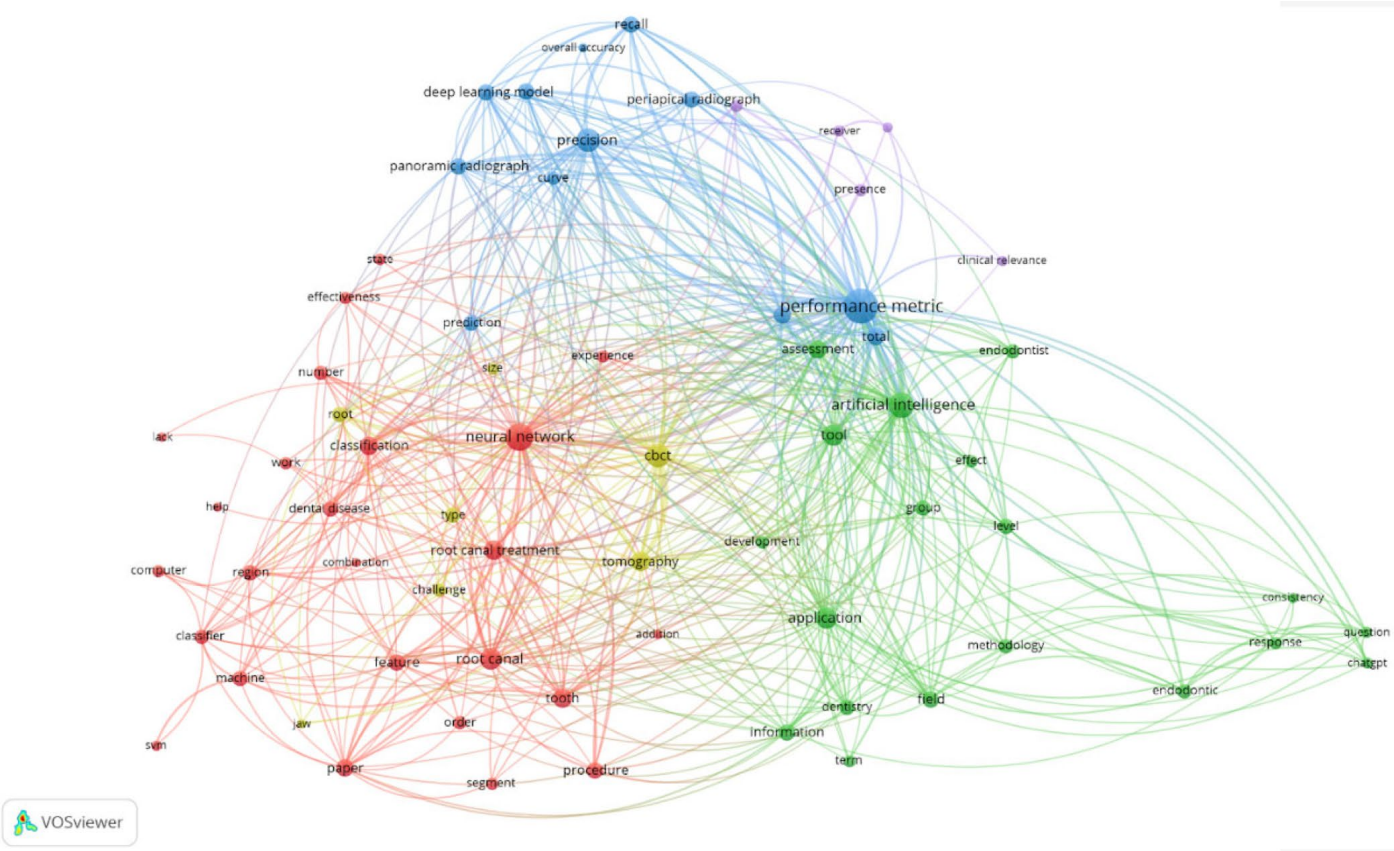
Co‐occurrence network of keywords. Each node represents an individual keyword, with colour and size indicating thematic clustering and frequency of occurrence. Connections between nodes reflect co‐occurrence relationships, with thicker connectors indicating stronger association.

‘Neural network’ and ‘artificial intelligence'’ were the most prominent terms. These were followed by ‘performance metrics’, a grouped category encompassing terms such as performance, reliability, and validity, and CBCT’ as a central, highly prevalent keyword. There are 5 clusters identified. The blue cluster includes terms such as ‘precision’, ‘periapical radiographs’, ‘panoramic radiographs'’ and recall', reflecting a significant focus in the literature on evaluating AI performance in assessing the endodontic outcomes across different imaging modalities. Other clusters reflect similar thematic characteristics, such as the green cluster being testing accuracy, and the red cluster being diagnostic classification (Figure 6).

**FIGURE 7:**
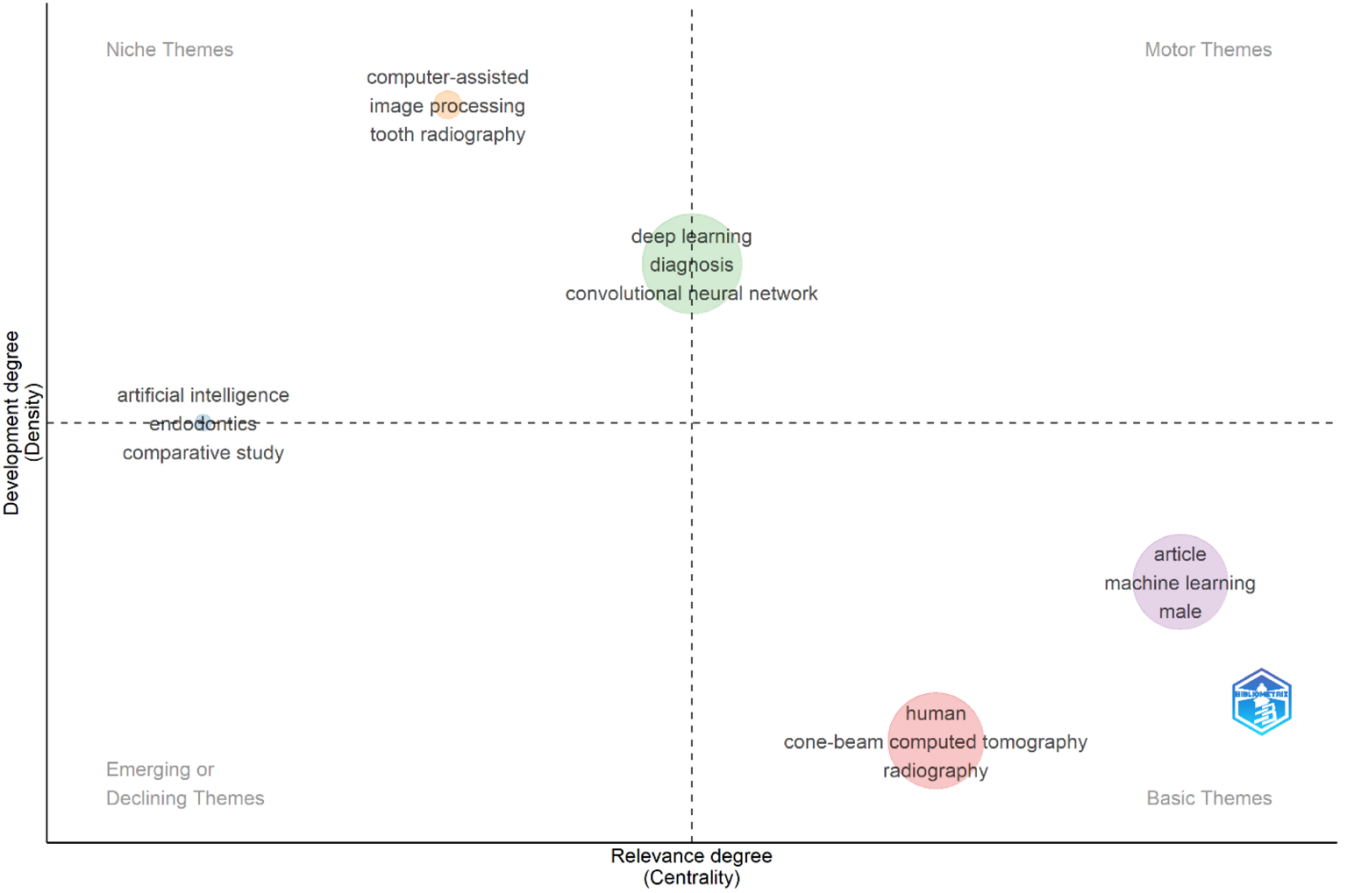
Thematic analysis showing regions of niche, motor, emerging, declining and basic themes.

According to Bibliometrix, the most relevant keywords that were not AI or endodontics were human' (*n* = 223), CBCT' (*n* = 102), radiography' (*n* = 51), and diagnostic imaging' (*n* = 48), which suggested a high emphasis on the utilization of AI in the interpretation and manipulation of dental imaging (Table S4). In addition, thematic analysis showed that both ‘deep learning’ and ‘convolutional neural networks’ in relation to ‘diagnosis’ remain central to the relevance, with ‘image processing’ considered a niche theme and ‘machine learning’ and ‘CBCT’ as basic themes (Figure 7).

### 3.5 Citations

The most cited work was by Ekert et al. [11], ‘Deep Learning for the Radiographic Detection of Apical Lesions’ with 256 citations. Hiraiwa et al.'s [2] study, ‘A Deep‐Learning Artificial Intelligence System for Assessment of Root Morphology of the Mandibular First Molar on Panoramic Radiography’, and Orhan et al.'s [12] publication, Evaluation of Artificial Intelligence for Detecting Periapical Pathosis on Cone‐Beam Computed Tomography Scans, both had 212 citations each.

The top three studies highlighted a clear emphasis in research on AI's diagnostic capabilities in radiographic imaging. Despite the explosive and exponential growth in publications from 2020 to 2024, nine of the top ten articles predate 2022. This could be explained by the fact that while these foundational studies dominate the field, more recent literature may have yet to reach its full academic impact or may still be in the early stages of academic recognition (Table 4).

## 4 Discussion

This bibliometric analysis provides the most comprehensive overview to date of artificial intelligence (AI) research within endodontics, incorporating 220 articles and reviewing the combined works of 47 countries, 444 institutions, and 957 authors. Although there were substantial quantities of articles produced, there appeared to be a limited amount of collaboration occurring on the authorial, institutional, and geographical levels. On the authorial level, 957 individual authors contributed to the analysed research, but wider collaborative research appeared to be less common. Rather, researchers appeared to produce publications in tighter clusters that were isolated from one another. Only 12 major groups of researchers (at least 2 distinct instances of co‐authorship) could be considered the predominant drivers behind the recent state of research in this field. Geographical proximity was a recurring feature within these individual isolated groups and was likely a major formative factor for these collaborative arrangements. The most prominent author within this field, Orhan, showed consistent collaborations with authors Çelik, Ö & Ezhov, M, albeit in distinct research groups, which led to notable publications, including Bayrakdar et al., 2022 and Orhan et al., 2020 [12, 27], respectively, the latter article having the third highest citation count among the analysed articles.

Orhan's contributions within this field revolved entirely around the broader research focus of two‐dimensional (2D) and three‐dimensional (3D) image analysis using deep learning AI models for application within clinical endodontics, consistent with the trending research climate throughout the past two decades. The largest collaboration network with consistent co‐authorship over multiple publications included authors Rey, D, Bianchi, J, Ruellas, A, Alvarez, MA and Rios, H, who focused specifically on 3D dental radiographic image segmentation using AI [32]. Similar trends followed at the institutional level, with most of the interest in this field of research primarily being driven by 10 distinct collaborative groups of institutions (at least 2 distinct instances of collaboration), also mostly bound by geographical location. On an international scale, the research was primarily driven by China, the United States, Turkey, and India, with notable publications including Li et al. [16], Setzer et al. [13], Ezhov et al. [15] and Sherwood et al. [29] respectively.

Of these countries, articles with affiliations to the United States had, on average, the highest citation counts (Highest TC = 212) [12], while publications associated with Indian institutions had the lowest (Highest TC = 45) [29]. Most published works by these countries were single‐country publications with no foreign collaborators except for the United States, with 70% of its total publications being multi‐national publications. However, a strong collaborative network between China and the United States, and between the United States and Turkey was visualised, likely attributable to the United States and China being the top two global leading figures in AI research since 2000 [33]. Difficulties regarding international collaboration, including language barriers and working around certain logistical hurdles, may have been a major contributing factor to the seemingly sparse number of global partnerships within this field of research. However, the bibliometrics had a lack of recognition of Taiwan (the Republic of China), which is grouped under ‘China’ in VOSViewer. Bibliometrix correctly identified 9 studies under Chung Yuan Christian University, which was the second highest ranked affiliation based in Taoyuan, Taiwan, yet the analysis did not appear to make a geographical distinction to China (Table S2). As well, one of the major network collaborations showed nodes between Chung Yuan Christian University, Ming Chi University of Technology and Chang Gung Memorial Hospital: all of which are universities in Taiwan. Irrespective of the ambiguous and controversial geopolitical recognition, such oversights would simultaneously over‐represent China (People's Republic of China) in their dominance in the field and under‐represent Taiwan.

The most frequently occurring keywords were analysed to determine the trend topics within this field of research over the years. The idea of computer‐aided diagnosis' within the field of endodontics has been researched since the past decade, with interest in this objective being shown from 2013 onwards. Notable emerging keywords between 2015–2017 include ‘three dimensional imaging' and computerized’ tomography, which indicated a shift towards the use of 3D‐imaging modalities within endodontic treatment and treatment planning [19, 25]. Notable keywords that emerged from 2021–2025 include image segmentation, ‘convolutional neural networks, ‘cone‐beam computerized tomography, deep learning, diagnostic imaging, and artificial intelligence, displaying exponential growth in appearance frequency until the present day. These changes in the research trend aptly describe the evolution of the ongoing investigation of the applications of AI within the field of endodontics, as the most commonly occurring theme within the analysed works was the development or modification of deep learning AI models for accurate assistance in endodontic diagnosis and treatment planning [34, 35].

The emergence of *education* as a distinct thematic cluster in 2024 likely reflects the widespread release and adoption of generative artificial intelligence tools, most notably large language models such as ChatGPT. It is important to distinguish this trend from earlier artificial intelligence research in endodontics, which has been dominated by traditional approaches, particularly computer vision models applied to image analysis tasks such as lesion detection and root morphology assessment. Generative artificial intelligence represents a fundamentally different class of technology, with primary applications in language‐based interaction, content generation and educational support, and with distinct limitations, risks and evaluation frameworks compared with image‐based models. This remains an emerging research area with limited empirical validation, and robust evidence regarding educational effectiveness, accuracy, and safety is still lacking.

Overall, the findings indicated that there is a current exponentially increasing interest in the use of AI in endodontics, particularly in association with the use of dental imaging methodologies, such as CBCT imaging, beginning from 2020 to 2021. At present, the most popular focus is on the use of AI in the form of deep learning systems involving neural networks, particularly the use of convolutional neural networks (CNNs). CNNs have gained increasing interest in the broader medical field and radiology for analysing visual data [36]. The shift in dominant keywords, from general descriptors such as *software* and *computer‐aided diagnosis* to specialised terms such as *deep learning*, *CBCT*, *CNNs*, and *image segmentation*, underscores a maturation of the field. These transitions reflect increasing confidence in AI's capacity to support radiographic interpretation, particularly in diagnosing apical periodontitis, mapping complex canal systems, and identifying anatomical variations relevant to treatment planning. Notably, the most cited works in this study were those validating CNN performance for periapical lesion detection [37, 38] and root morphology assessment [20], highlighting the centrality of imaging‐driven diagnostics to AI research in endodontics.

In clinical endodontics, this has been shown with significant reoccurrences as a popular keyword appearing in the extracted studies and significant associations to other popular keywords such as CBCT and, to a lesser extent, to other imaging modalities such as panoramic and periapical radiographs. Limited FOV CBCT scans have become increasingly more popular with endodontists, and the research landscape currently suggests that there is a thought that AI may assist practitioners with diagnosis and detection of apical pathology and improved visualisation and assessment of root morphology.

Indeed, the three most cited articles all involved evaluating the use of deep learning systems in association with dental imaging, with Ekert et al. [11] evaluating the discriminatory ability of a single CNN for detection of apical pathology from panoramic radiographs, Hiraiwa et al. [2] assessing the performance of a deep learning system utilising a CNN to assess distal root morphology of the mandibular first molar from panoramic radiography, and Setzer et al. [13] describing the performance of a CNN to detect periapical lesions in CBCT imaging. Current research appeared to be partly inspired by the findings of these studies, evidenced by the increasing appearance of image segmentation, ‘convolutional neural networks, “cone‐beam computerized tomography, deep learning, diagnostic imaging” and artificial intelligence’ keywords.

To minimise any effects of selection bias, multiple databases (Scopus, PubMed, EMBASE) were accessed, and articles were indiscriminately selected regardless of their country of publication. Three articles were excluded due to being non‐English. As English is the common language of science, articles indexed in English are more likely to be to be accessible and widely disseminated. Hence, the decision to limit the analysis to English‐language articles was made in consideration of database accessibility and consistency [39]. In addition to this, 2 articles from the EMBASE database that were not available on either the Scopus or PubMed databases were excluded during analysis with Bibliometrix, due to software‐specific limitations. However, following this methodology allowed for a metadata analysis of only a single database, which reduces the need for data harmonisation and also citation count discrepancies in different databases. It should be noted that no standardised protocol exists for the interpretation of bibliometric data, and thus, while this study aimed to provide an objective overview of this research landscape, individual bias may ultimately create slight differences in the conclusions drawn from the results. Previous work have suggested the use of the BIBLIO Checklist to standardise reporting, but the suggested framework is still in the preliminary stages of development [40]. Due to resource limitations, Web of Science was not included as a database but could be considered to reduce database‐related bias in future bibliometric studies involving the use of Bibliometrix, with its metadata being preferential to Scopus (https://www.bibliometrix.org/home/index.php/faq). It is difficult to ascertain precisely which metric determines the level of impact, and as such, this study accounted for multiple measures such as total citations (aggregate or per year), h‐index, authorship (aggregate and fractionalised). Given the rate of production of research in this field, regular bibliometric review with similar methodology is required to track the changes in trends over the years, especially in an emerging science. Furthermore, while bibliometric analyses were powerful in identifying trends and connections, they do not allow for direct causal conclusions about the impact of AI on clinical outcomes in endodontics.

There are several promising research directions that are less addressed in the current literature and can be the subject of future investigations. First, multimodal AI models that integrate radiographic imaging with clinical parameters (e.g., pulp vitality tests or patient symptoms) and demographic data could enhance diagnostic accuracy and clinical decision‐making beyond what image‐only models can achieve. Second, federated learning offers a privacy‐preserving framework for multi‐centre collaboration, enabling institutions to collectively train robust AI models without sharing sensitive patient data, which is a critical consideration given the geographic fragmentation of research collaboration identified in this analysis. Third, while current research predominantly focuses on diagnostic applications, there is significant potential for treatment outcome prediction and personalised treatment planning using longitudinal datasets that track patients from initial diagnosis through post‐treatment follow‐up. Fourth, the challenge of limited dataset sizes in endodontic AI could be addressed through advanced training paradigms, including self‐supervised pre‐training using unlabelled dental images to learn generalisable data representations, or synthetic data augmentation using generative models such as diffusion‐based architectures to expand training datasets while preserving anatomical realism. Finally, cross‐modality AI conversion, such as generating pseudo‐3D reconstructions from 2D periapical radiographs or enhancing CBCT images to approximate micro‐CT resolution, could democratise access to advanced diagnostic information in clinical settings where high‐resolution imaging is unavailable.

## 5 Conclusions

The field of AI in endodontics is experiencing rapid academic growth, particularly in the application of deep learning systems utilising CNNs and dental imaging analysis, with minor interest in the educational performance of AI chatbots. While collaboration remains limited at the authorial, institutional, and global levels, the recent explosive growth of interest suggests there is likely to be an increased volume of research and room for more varied focus in integrating AI into endodontic practice beyond imaging analysis.

## Conflicts of Interest

The authors declare no conflicts of interest.

## Supporting information


**Table S1:** Search string used for respective database

**Table S2:** Top 25 university affiliations ranked by journal article production

**Table S3:** Journal rankings by h‐index, g‐index, m‐index, total citations (TC), publication counts (NP) and publications year start (PY‐start)

**Table S4:** Most frequent words identified in Bibliometrix.

## Data Availability

The data that support the findings of this study are available from the corresponding author upon reasonable request.
