# Supplementary material for: Artificial Intelligence in Endodontics—A Bibliometric Analysis
Source: Aust Endod J. 2026 Apr 21;52(2):619–29. doi: 10.1111/aej.70080 (PMC13436042; doi:10.1111/aej.70080)
Supplement: Supplementary file 1 [file AEJ-52-619-s001.docx]

| **Supplementary Table S2**. Top 25 university affiliations ranked by journal article production  **Affiliation** | **Articles** |
| --- | --- |
| Ankara University | 16 |
| Chung Yuan Christian University | 9 |
| Islamic Azad University | 8 |
| University Of Pennsylvania | 8 |
| Peking University School And Hospital Of Stomatology | 7 |
| Shandong University | 7 |
| Tongji University | 7 |
| Wuhan University | 7 |
| Hanoi Medical University | 6 |
| Indian Institute Of Technology Indore | 6 |
| Near East University | 6 |
| Sichuan University | 6 |
| University Of Michigan | 6 |
| Eskisehir Osmangazi University | 5 |
| Medical University Of Graz | 5 |
| Sun Yat-Sen University | 5 |
| Temple University | 5 |
| University Of North Carolina | 5 |
| Yonsei University | 5 |
| Yonsei University College Of Dentistry | 5 |
| Aichi-Gakuin University School Of Dentistry | 4 |
| Ateneo De Manila University | 4 |
| Eskişehir Osmangazi University | 4 |
| Gazi University | 4 |
| Hamadan University Of Medical Sciences | 4 |
