# Supplementary material for: Artificial Intelligence in Endodontics—A Bibliometric Analysis
Source: Aust Endod J. 2026 Apr 21;52(2):619–29. doi: 10.1111/aej.70080 (PMC13436042; doi:10.1111/aej.70080)
Supplement: Supplementary file 2 [file AEJ-52-619-s002.docx]

**Supplementary Table S1.** Search string used for respective database

| PubMed: | ("Endodontics"[Mesh] OR "dental pulp"[tiab] OR pulpotomy[tiab] OR pulpectomy[tiab] OR pulpitis[tiab] OR endodont*[tiab] OR pulpally[tiab] OR pulpal[tiab] OR "root canal"[tiab] OR periapical[tiab] OR "pulp capping"[tiab] OR "vital pulp"[tiab]) AND ("machine learning" [MeSH] OR "machine learning"[tiab] OR "Artificial Intelligence" [tiab] OR "artificially intelligent" [tiab] OR "Artificial Intelligence" [MeSH] OR "deep learning" [tiab] OR "neural network*" [tiab] OR "neural networks, computer" [MeSH] OR "intelligent machine*" [tiab] OR ai[tiab] OR robot[tiab] OR "neural network"[tiab] OR "expert system"[tiab]),  **452 records identified; 10 duplicates; 442 added to screening.** |
| --- | --- |
| EMBASE: | ('endodontics'/exp OR 'dental pulp':ti,ab OR pulpotomy:ti,ab OR pulpectomy:ti,ab OR pulpitis:ti,ab OR endodont*:ti,ab OR pulpally:ti,ab OR pulpal:ti,ab OR 'root canal':ti,ab OR periapical:ti,ab OR 'pulp capping':ti,ab OR 'vital pulp':ti,ab) AND ('machine learning'/exp OR 'machine learning':ti,ab OR 'artificial intelligence':ti,ab OR 'artificially intelligent':ti,ab OR 'artificial intelligence'/exp OR 'deep learning':ti,ab OR 'neural network*':ti,ab OR 'neural network'/exp OR 'intelligent machine*':ti,ab OR ai:ti,ab OR robot:ti,ab OR 'expert system':ti,ab)  **478 records identified; 369 duplicates; 109 added to screening** |
| Scopus | ( TITLE-ABS-KEY ( artificial AND intelligence ) OR TITLE-ABS-KEY ( ai ) OR TITLE-ABS-KEY ( machine AND learning ) OR TITLE-ABS-KEY ( deep AND learning ) OR TITLE-ABS-KEY ( robot ) OR TITLE-ABS-KEY ( neural AND network ) OR TITLE-ABS-KEY ( expert AND system ) OR TITLE-ABS-KEY ( intelligent AND machine ) OR TITLE-ABS-KEY ( expert AND system ) ) AND ( TITLE-ABS-KEY ( endodontics ) OR TITLE-ABS-KEY ( root AND canal AND therapy ) OR TITLE-ABS-KEY ( root AND canal AND treatment ) OR TITLE-ABS-KEY ( endodontic AND treatment ) OR TITLE-ABS-KEY ( dental AND pulp ) OR TITLE-ABS-KEY ( pulpotomy ) OR TITLE-ABS-KEY ( pulpectomy ) OR TITLE-ABS-KEY ( pulpitis ) OR TITLE-ABS-KEY ( periapical ) OR TITLE-ABS-KEY ( pulp AND capping ) OR TITLE-ABS-KEY ( vital AND pulp ) ) AND  ( LIMIT-TO ( DOCTYPE , "ar" ) OR LIMIT-TO ( DOCTYPE , "English" ) )  **1,535 records identified; 1,094 duplicates; 441 added to screening.** |
