# Supplementary material for: Artificial Intelligence in Endodontics—A Bibliometric Analysis
Source: Aust Endod J. 2026 Apr 21;52(2):619–29. doi: 10.1111/aej.70080 (PMC13436042; doi:10.1111/aej.70080)
Supplement: Supplementary file 3 [file AEJ-52-619-s003.docx]

**Supplementary Table S3.** Journal rankings by h-index, g-index, m-index, total citations (TC), publication counts (NP) and publications year start (PY-start)

| **Source** | **h-index** | **g-index** | **m-index** | **TC** | **NP** | **PY-start** |
| --- | --- | --- | --- | --- | --- | --- |
| Journal Of Endodontics | 9 | 20 | 0.529 | 700 | 20 | 2009 |
| Dentomaxillofacial Radiology | 8 | 10 | 0.235 | 537 | 10 | 1992 |
| Journal Of Dentistry | 7 | 10 | 1.75 | 195 | 10 | 2022 |
| International Endodontic Journal | 5 | 8 | 0.357 | 431 | 8 | 2012 |
| Clinical Oral Investigations | 4 | 8 | 0.308 | 115 | 8 | 2013 |
| Diagnostics | 4 | 6 | 1 | 52 | 6 | 2022 |
| Journal Of Clinical Medicine | 4 | 6 | 1.333 | 44 | 6 | 2023 |
| Scientific Reports | 4 | 7 | 0.8 | 172 | 7 | 2021 |
| Applied Sciences (Switzerland) | 3 | 3 | 0.5 | 29 | 3 | 2020 |
| Bmc Oral Health | 3 | 4 | 1 | 22 | 9 | 2023 |
