# Supplementary material for: Artificial Intelligence in Endodontics—A Bibliometric Analysis
Source: Aust Endod J. 2026 Apr 21;52(2):619–29. doi: 10.1111/aej.70080 (PMC13436042; doi:10.1111/aej.70080)
Supplement: Supplementary file 4 [file AEJ-52-619-s004.docx]

**Supplementary Table S4.** Most frequent words identified in Bibliometrix.

| **Words** | **Occurrences** |
| --- | --- |
| Human | 223 |
| Cone-Beam Computed Tomography | 102 |
| Artificial Intelligence | 93 |
| Deep Learning | 87 |
| Article | 60 |
| Radiography | 51 |
| Diagnostic Imaging | 48 |
| Procedures | 48 |
| Diagnosis | 47 |
| Endodontics | 39 |
